# Supplementary figures and images for: Rising Temperatures, Falling Leaves: Predicting the Fate of Cyprus’s Endemic Oak under Climate and Land Use Change
Source: Plants (Basel). 2024 Apr 16;13(8):1109. doi: 10.3390/plants13081109 (PMC11053427; doi:10.3390/plants13081109)

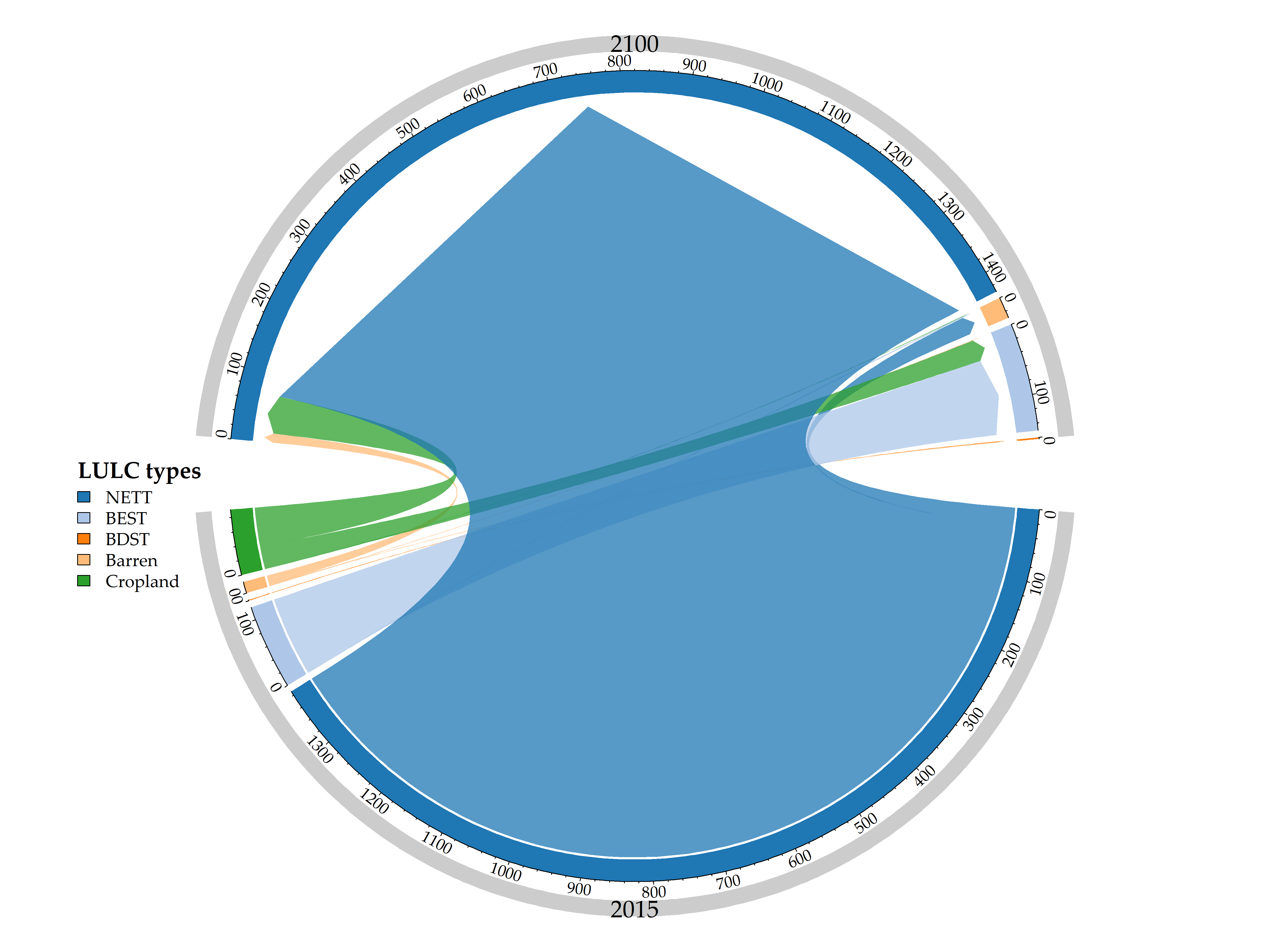

Supplement: Supplementary file 1 [file plants-13-01109-s001.zip › Figure S1.png]

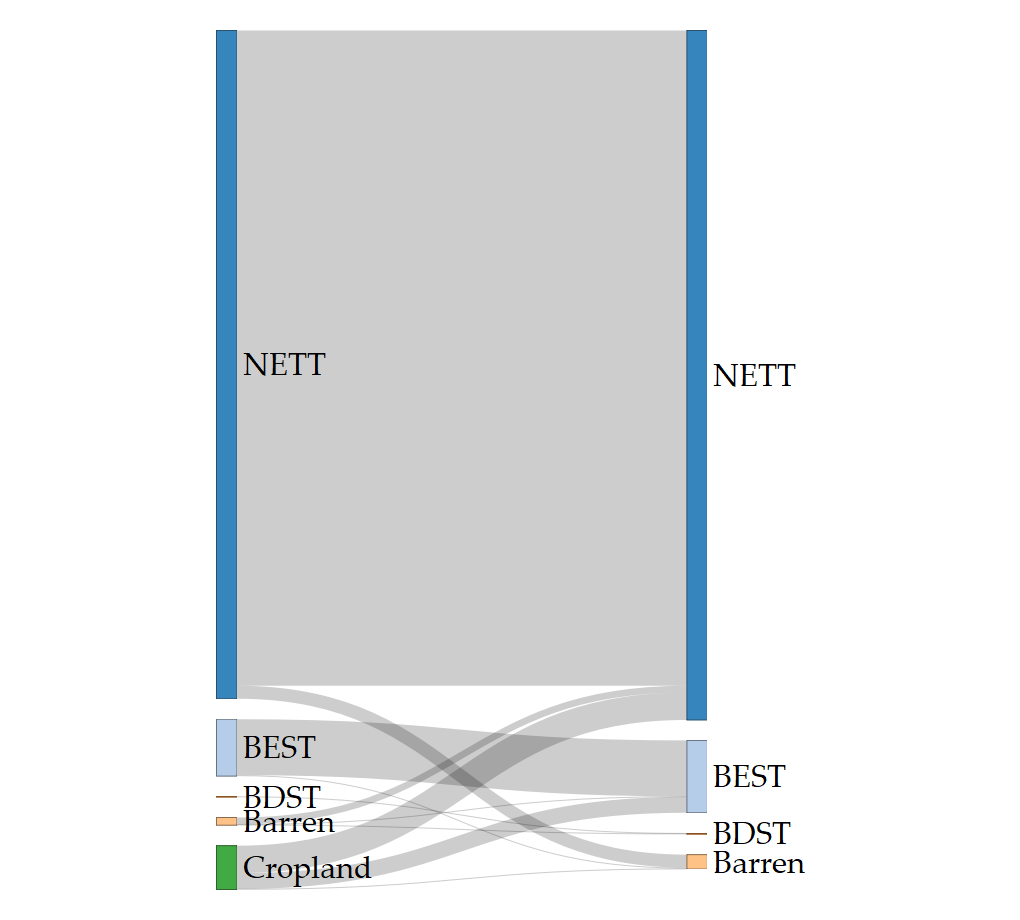

Supplement: Supplementary file 1 [file plants-13-01109-s001.zip › Figure S10.png]

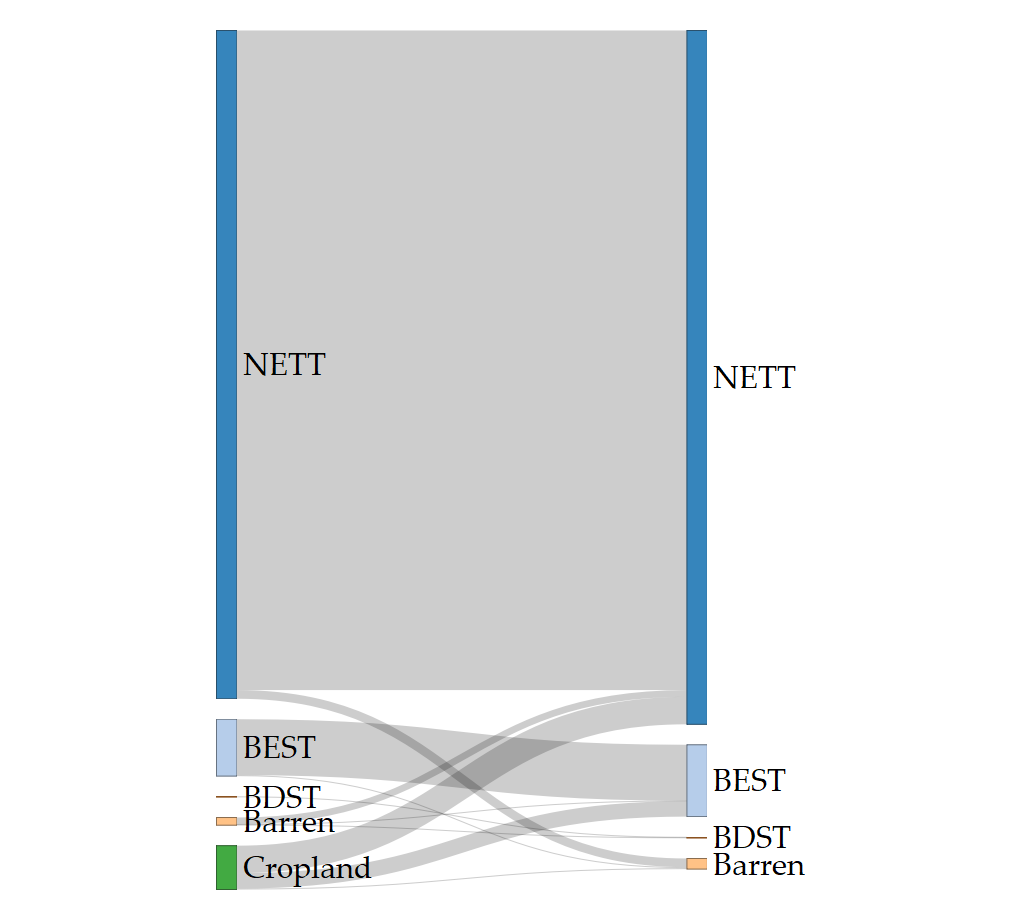

Supplement: Supplementary file 1 [file plants-13-01109-s001.zip › Figure S11.png]

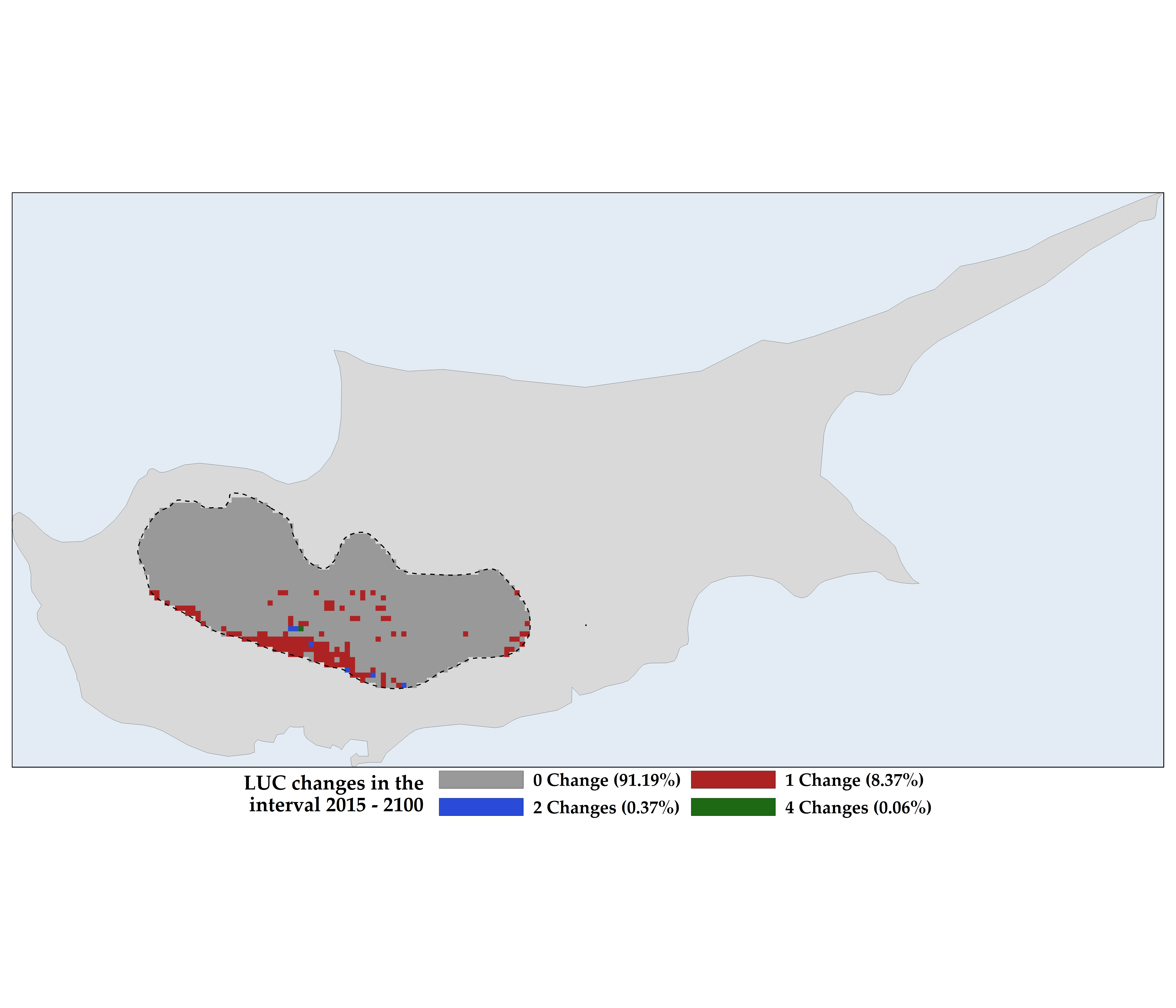

Supplement: Supplementary file 1 [file plants-13-01109-s001.zip › Figure S12.png]

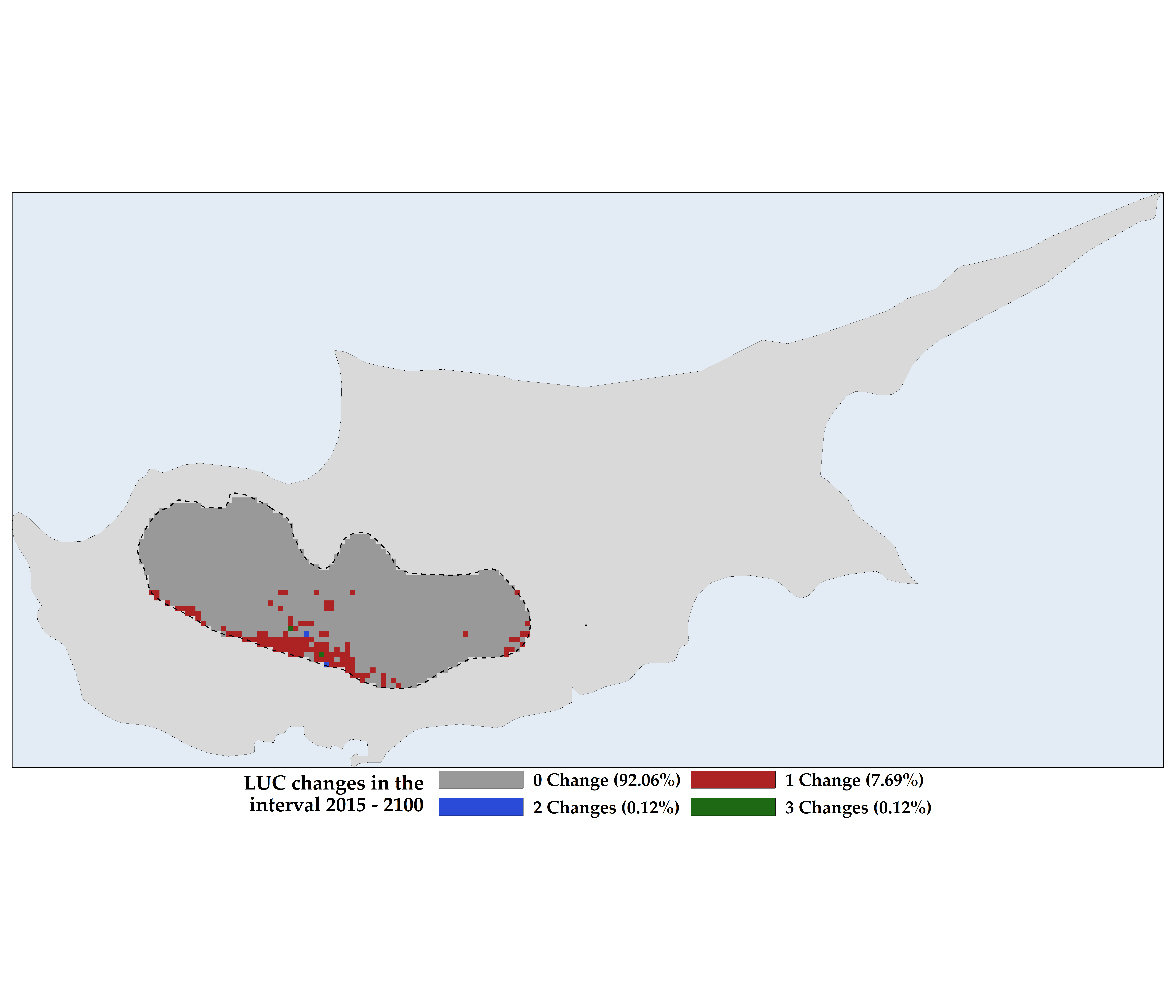

Supplement: Supplementary file 1 [file plants-13-01109-s001.zip › Figure S13.png]

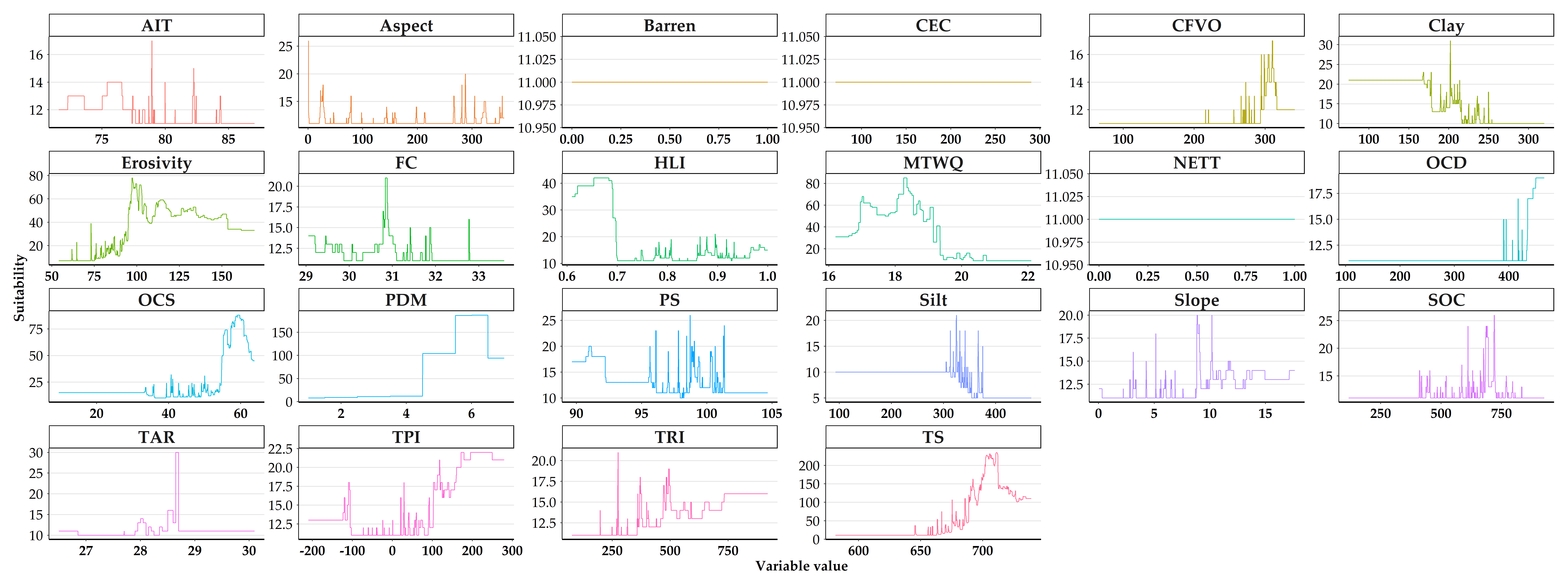

Supplement: Supplementary file 1 [file plants-13-01109-s001.zip › Figure S14.png]

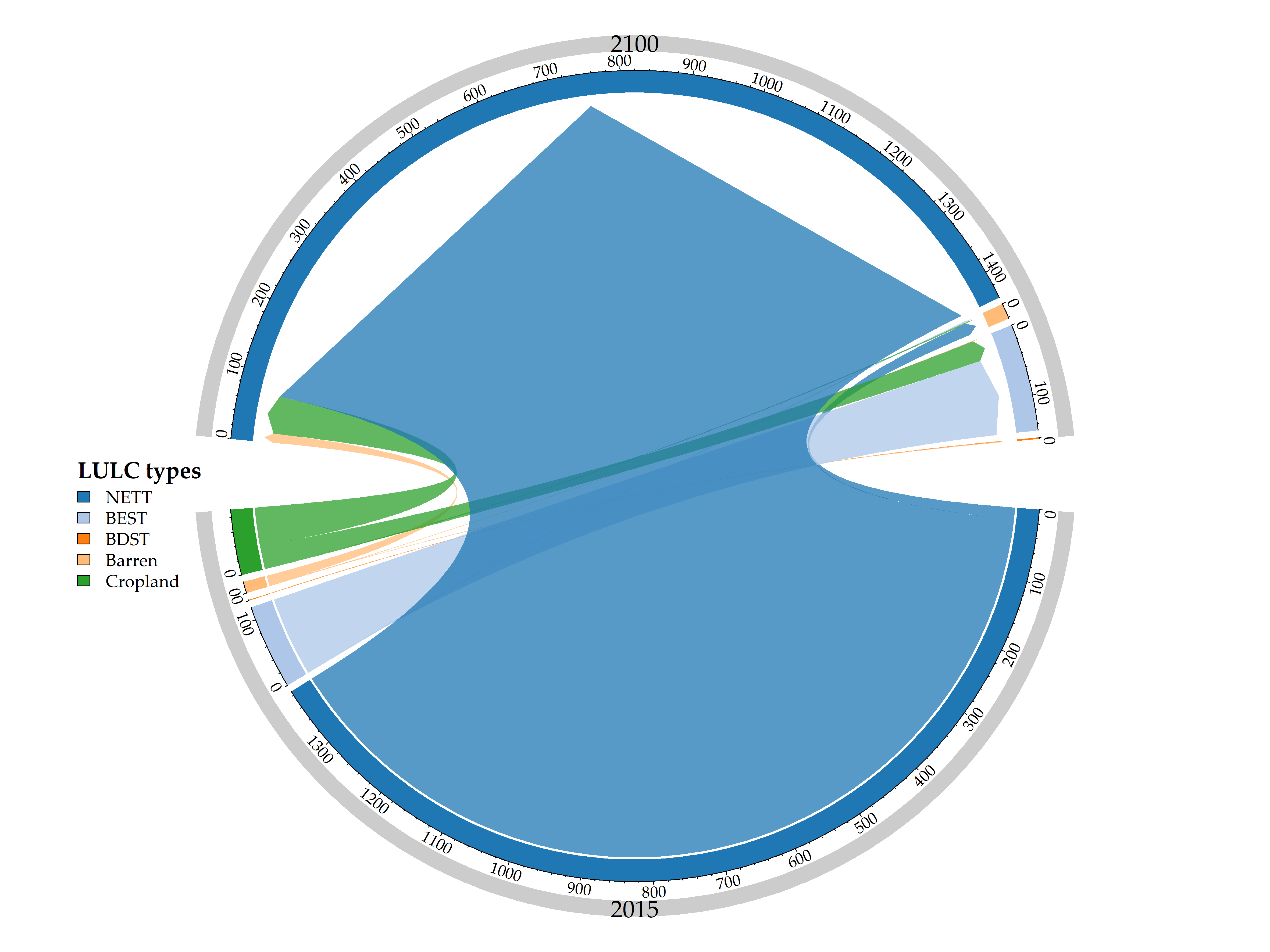

Supplement: Supplementary file 1 [file plants-13-01109-s001.zip › Figure S2.png]

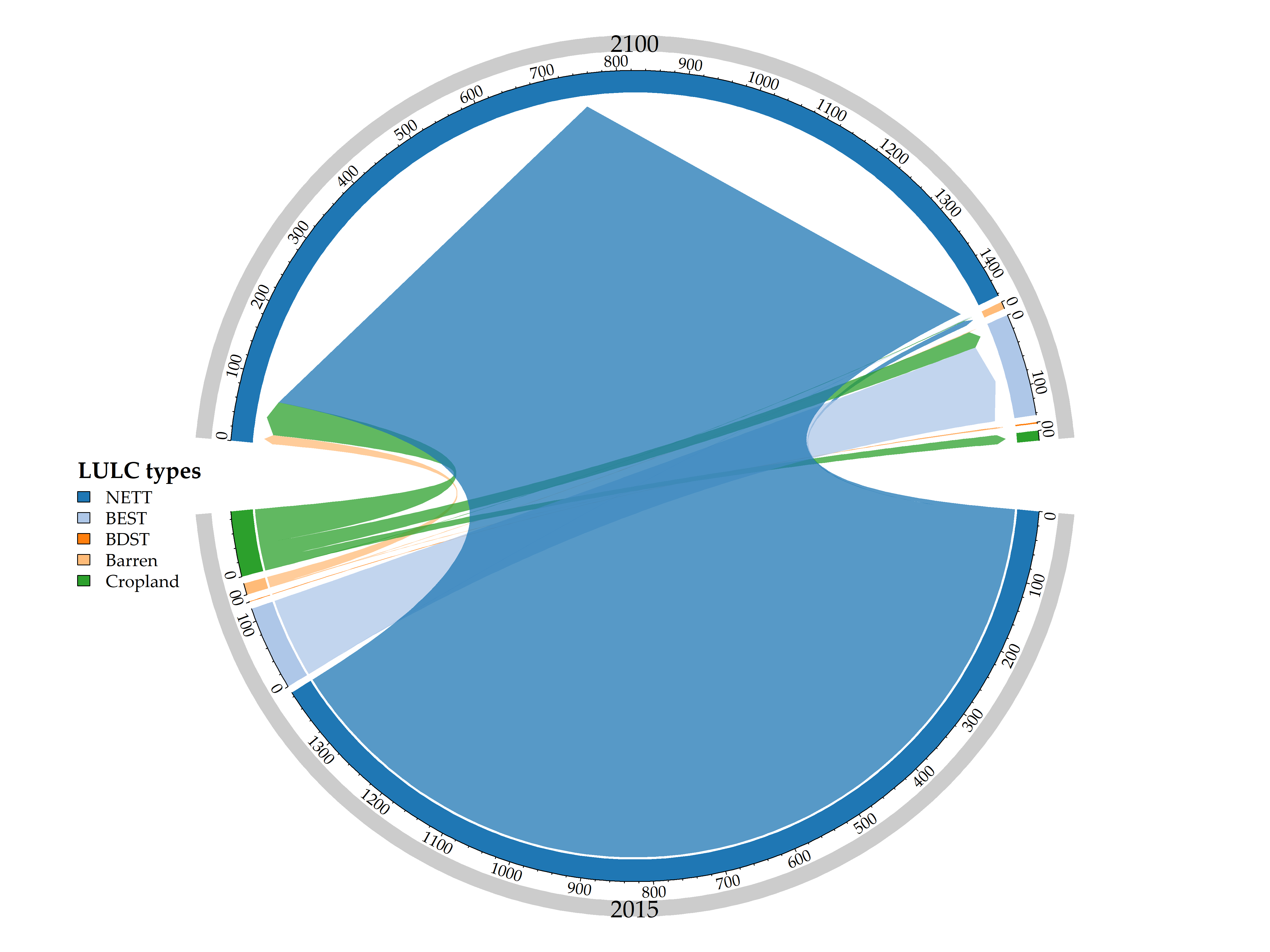

Supplement: Supplementary file 1 [file plants-13-01109-s001.zip › Figure S3.png]

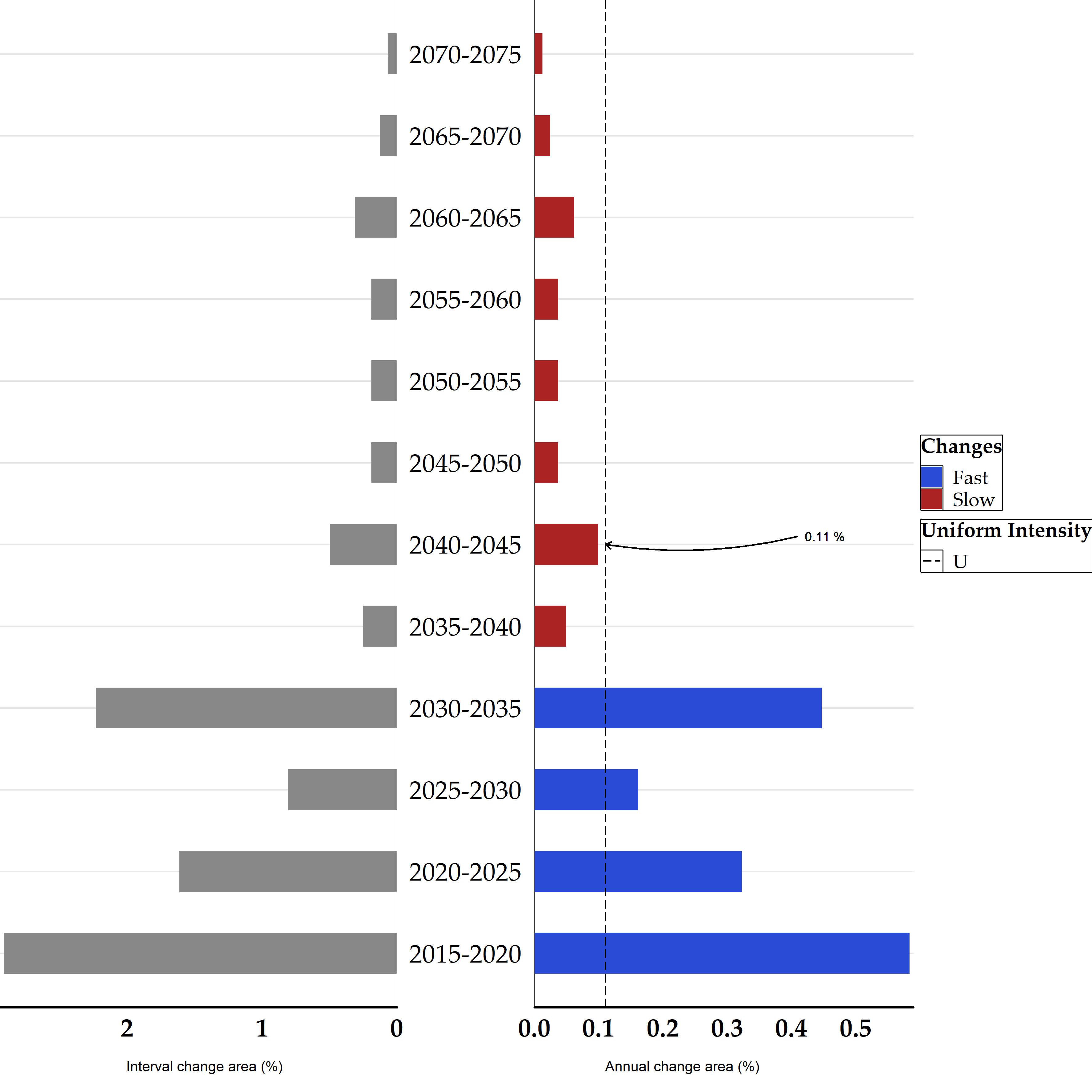

Supplement: Supplementary file 1 [file plants-13-01109-s001.zip › Figure S4.png]

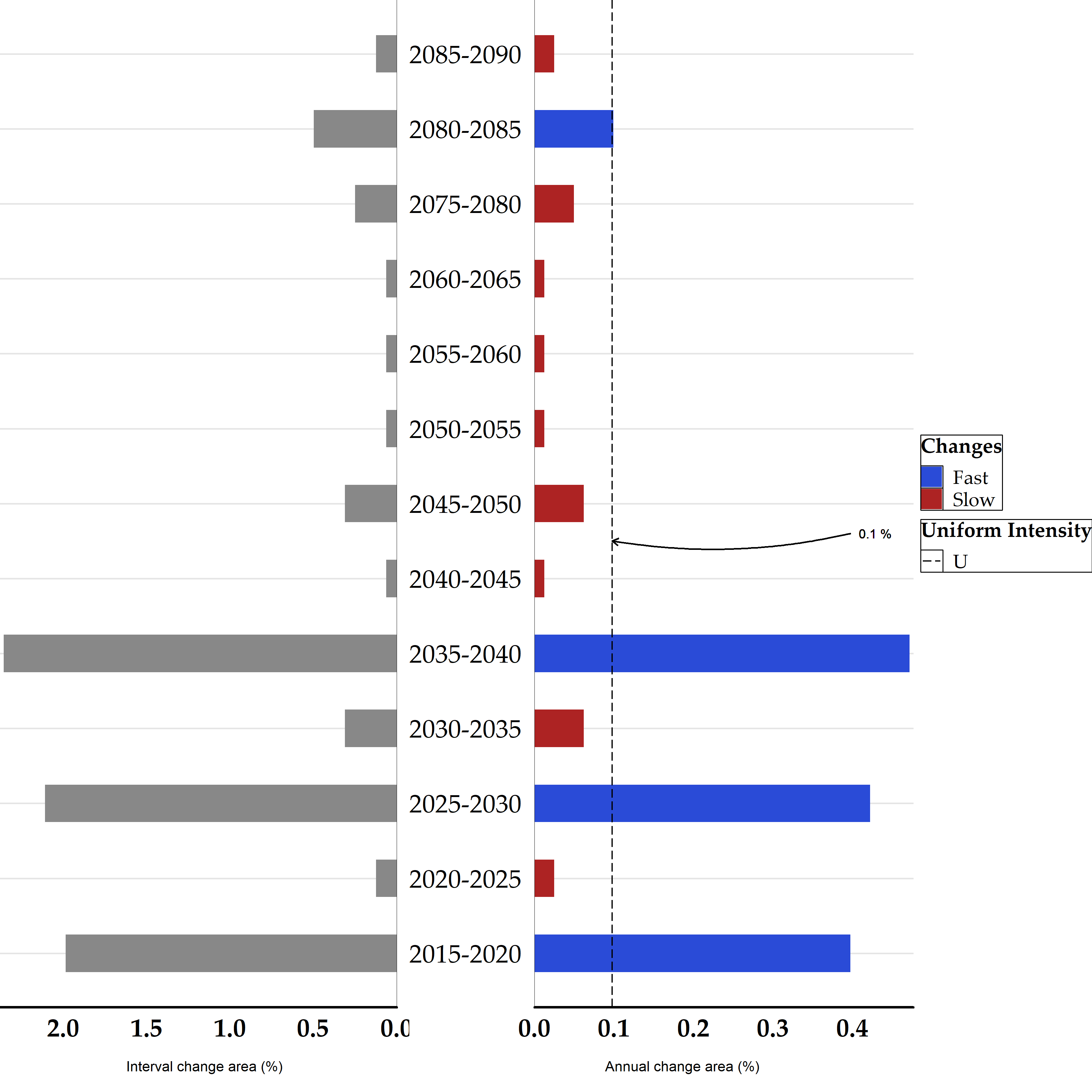

Supplement: Supplementary file 1 [file plants-13-01109-s001.zip › Figure S5.png]

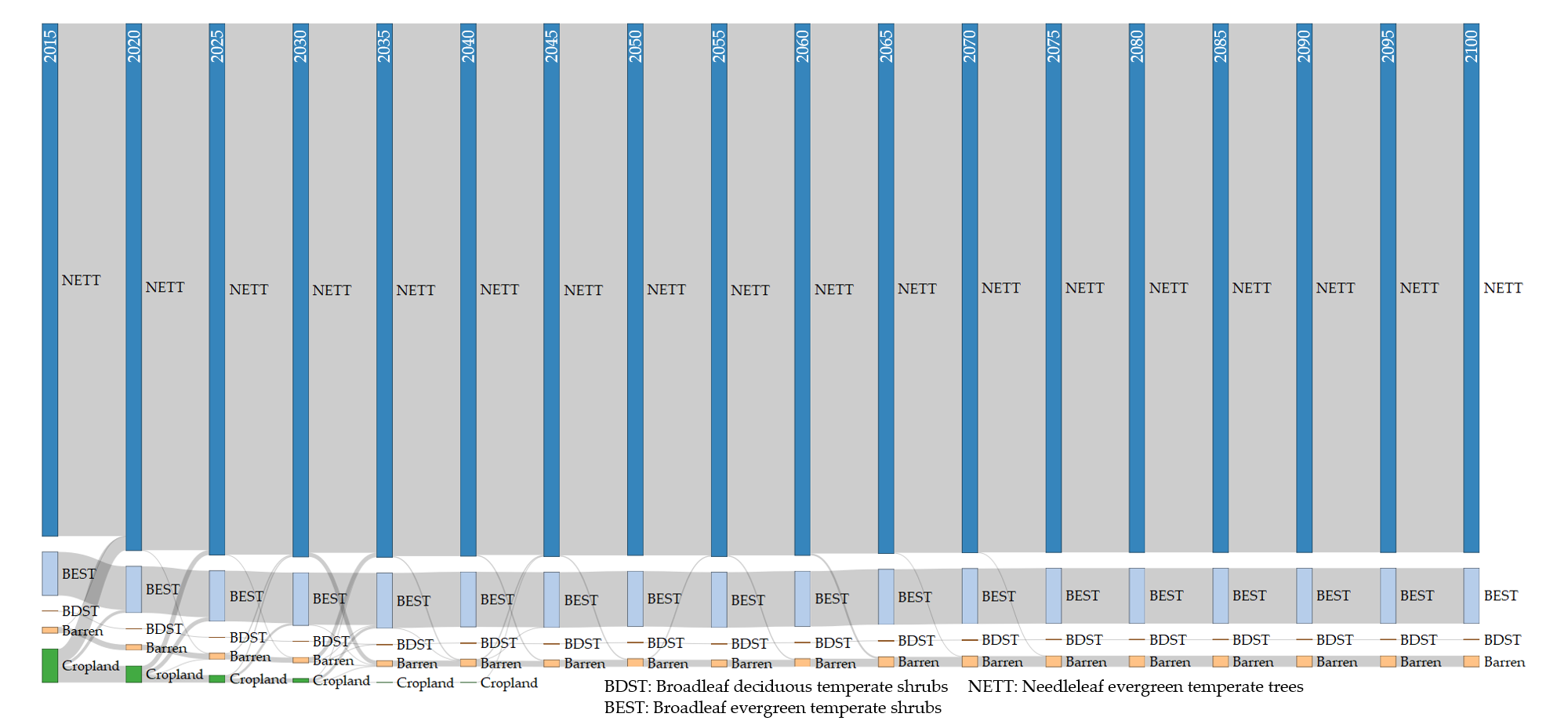

Supplement: Supplementary file 1 [file plants-13-01109-s001.zip › Figure S7.png]

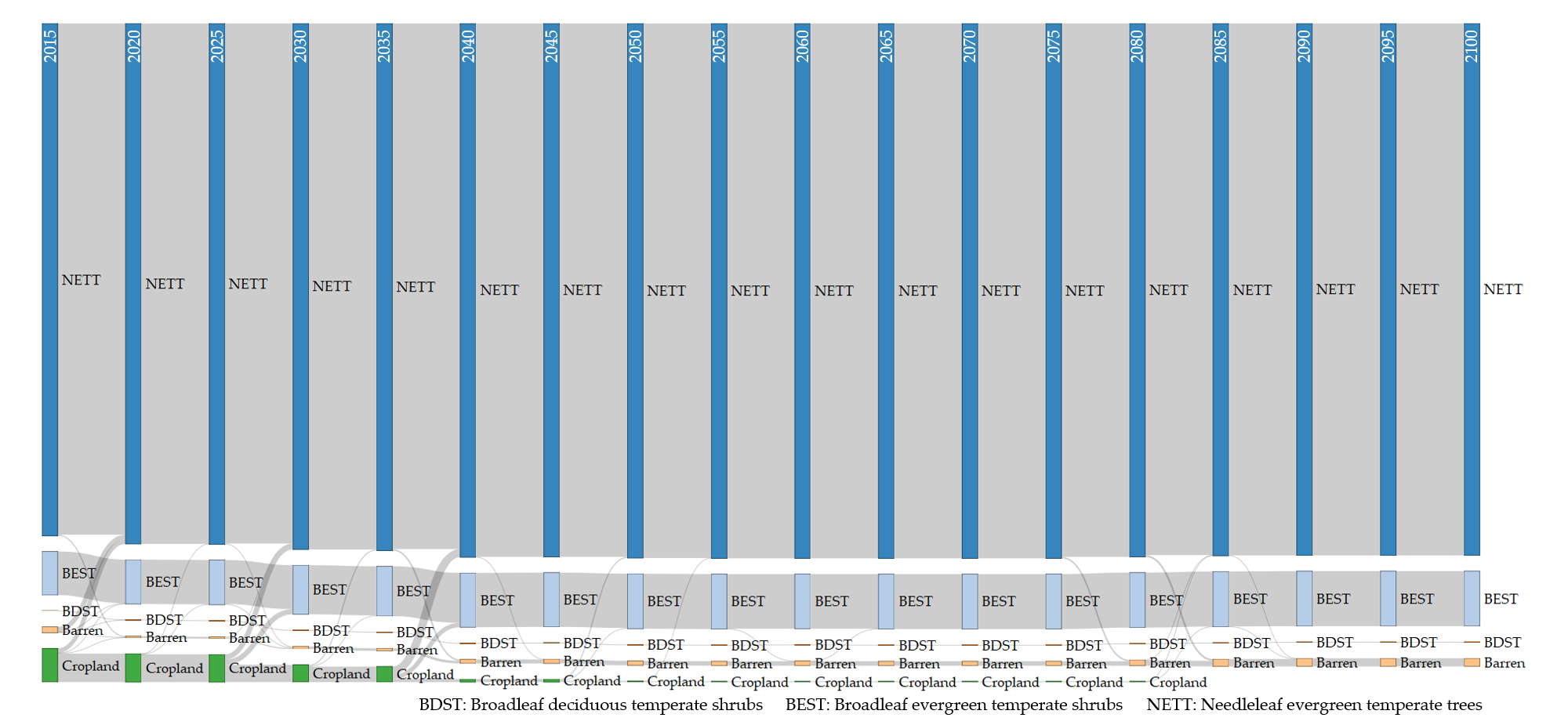

Supplement: Supplementary file 1 [file plants-13-01109-s001.zip › Figure S8.png]

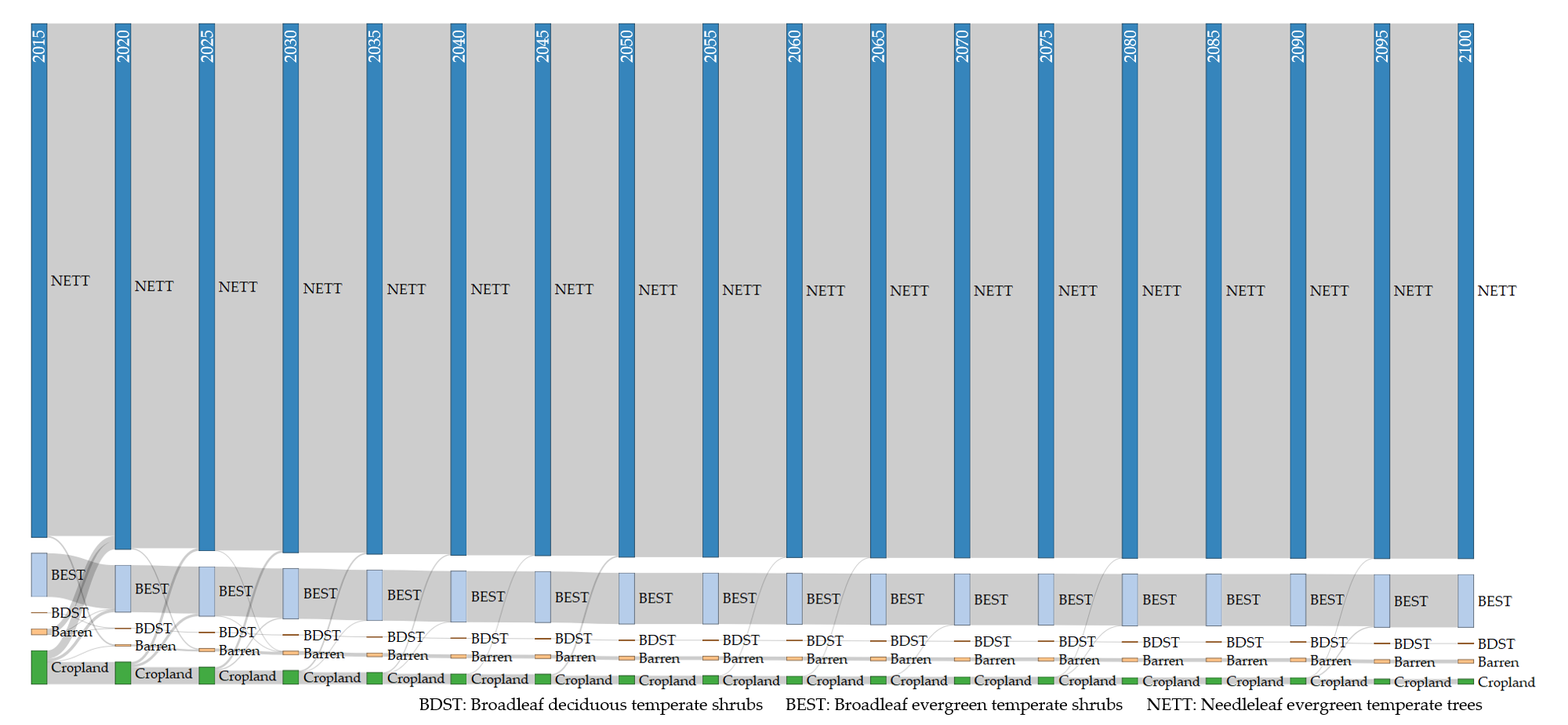

Supplement: Supplementary file 1 [file plants-13-01109-s001.zip › Figure S9.png]
